# Volatile Profile of Nuts, Key Odorants and Analytical Methods for Quantification

**DOI:** 10.3390/foods10071611

**Published:** 2021-07-12

**Authors:** Arantzazu Valdés García, Raquel Sánchez Romero, Adriana Juan Polo, Soledad Prats Moya, Salvador E. Maestre Pérez, Ana Beltrán Sanahuja

**Affiliations:** Analytical Chemistry, Nutrition and Food Sciences Department, University of Alicante, P.O. Box 99, E-03080 Alicante, Spain; r.sanchez@ua.es (R.S.R.); adriana.juan@ua.es (A.J.P.); maria.prats@ua.es (S.P.M.); salvador.maestre@ua.es (S.E.M.P.); ana.beltran@ua.es (A.B.S.)

**Keywords:** nut, volatile, aroma, flavor, key odorants, analytical methods, solid-phase microextraction

## Abstract

The presence of nuts in diets has notably increased due to their composition, and the presence of antioxidants and their unsaturated fatty acid profile has led to a considerable increase in their consumption. The volatile profile of nuts is important from different points of view. It affects consumer’s selection, influences raw material selection for the production of composite foods, dictates variety selection in breeding programs, and, from a quality perspective, its changes can indicate food degradation or alteration. A review of the published bibliography concerning the determination of volatiles in nuts has been carried out. The information retrieved has been divided into four main sections. First, a discussion on the main volatiles present in nuts is performed; next, a revision of the methods used to determine the volatiles is presented; and, finally, two sections describing how harvesting conditions, healthy state and the thermal treatment of nuts modifies their volatile profile are added. Analysis of the published bibliography denoted the complexity of volatile determination and the different variables that can modify the compounds present in the volatile fraction of nuts.

## 1. Introduction

Food odor and aroma have a great influence on consumers’ preference. These attributes are related to different volatile chemicals. Volatiles are a set of compounds with a relatively low molecular weight and high vapor pressure [1]. They include different classes of chemicals such as alcohols, hydrocarbons, esters, terpenes and aldehydes. The characterization of this complex and heterogeneous mix is important in food quality control and consequently, for the food industry [2].

Molecules with low molecular weight are perceived in the nose and mouth sensors faster than others with higher molecular weight and, for this reason, they condition the final flavor of the product. These predominant compounds are known as odorants, and they are very important as some of them are associated with pleasant odors, and others with off-odors. Study of the chemical composition of the volatile profile of foods allows us to understand the optimal conditions for food production and contributes to obtaining valuable information about their composition. Odor molecules arrive into the nose by air, and they are perceived by the olfactory mucosa via orthonasal smell. Meanwhile, aromas are perceived in the nose by the olfactory receptors, in the olfactory epithelium via the retronasal pathway, when the food is put into the mouth and the cells are broken by chewing, thus releasing volatile compounds. Odor and aroma perception is the result of the activation of odorant receptors generating complex signals that are sent to the central nervous system [3]. The amount and type of volatiles released from the food highly depends on the nature of the sample matrix and the state of the food [4]. More specifically, the entire volatile profile reflects the phenotypic or metabolic state of foods. In plants, volatile compounds have diverse and important functions such as to attract pollinators to flower organs, to protect damage plants from the attack of herbivores and to exchange some low molecular weight terpenes during light changes, droughts, or other stress situations for the plant [4].

Nuts are indehiscent dry fruits with one seed and a thick, hard pericarp. In the botanical sense, they are produced by some families of the order *Fagales* [5]. These families are the *Juglandaceae* (walnut and pecan nut), the *Fagaceae* (chestnut), and the *Betulaceae* (hazelnut). In the culinary sense, the term nut is applied to a wide variety of dried seeds and fruits and to any large, oily kernel found within a shell. These nuts belong to the *Fabaceae* (peanut), the *Rosaceae* (almond) and the *Anacardiaceae* (pistachio) families. From the food composition point of view, nuts are characterized by having a low water content, i.e., usually less than 50% weight, although some exceptions exist [6]. Due to the group’s diversity, nut volatile profiles are expected to be diverse.

**Figure 1:**
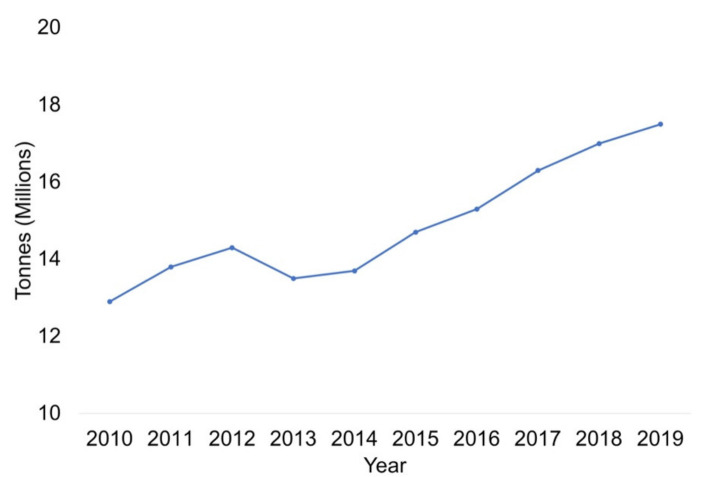
Worldwide nut production from 2010 to 2019.

Based on data recorded by the Food and Agriculture Organization of the United Nations (FAOSTAT), worldwide nut production has increased significantly in recent years, from 12.9 Mtons in 2010 up to 17.5 Mtons in 2019 (Figure 1) [7]. In 2019, California was the top worldwide producer of nuts, with an estimated annual production of 27.0% of the total. The United States was the second largest producer at around 17.4%, followed by Turkey (7.5%), Iran (4.9%), Côte d’Ivoire (4.6%), India (4.3%) and Spain (3.3%).

**Figure 2:**
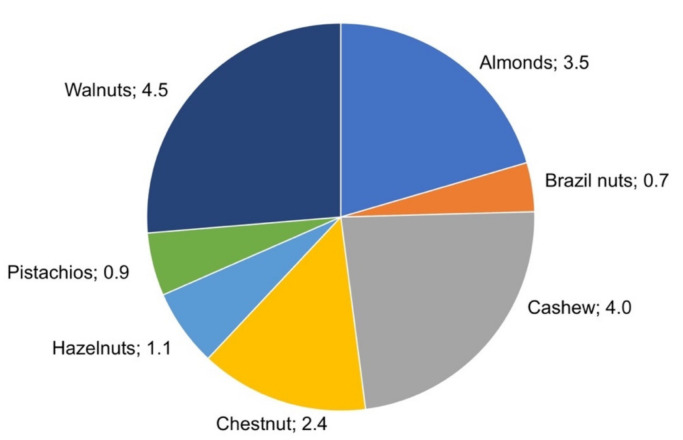
Worldwide nut production (Mtons) registered by FAOSTAT in 2019.

The majority of worldwide nut production in 2019 was registered as walnuts, followed by almonds, cashew, chestnut, hazelnuts, and finally, pistachio and Brazil nuts (Figure 2) [7]. Nuts are seeds showing a high sensory appeal and numerous health benefits due to their composition. In this sense, they present a high content in terms of protein (13–26% total weight) and lipids (43–76% total weight), carbohydrates (9–30% total weight) and fiber (7–12% total weight) with less than 6.4% water [6]. Nuts can be eaten raw, roasted, or salted as snacks. Thus, they are widely added to some important food formulations such as ice creams, chocolates, confectioneries, cookies, cereal bars and cakes. Due to their high level of dry matter and the possibility of undergoing technological processing, the volatile profile of nuts can undergo significant modifications compared to the profile of raw products.

Today, nuts are important foods whose presence in a healthy diet is recommended. They have relatively high levels of antioxidants and a fatty acid profile that is beneficial in reducing the risk of cardiovascular and degenerative diseases. However, differences have been reported due to their organoleptic properties. It is precisely the amount of certain monounsaturated and polyunsaturated acids that mainly condition their acceptability. This fact influences their flavor, as unsaturated fatty acids are prone to oxidation. Even though oxidative rancidity should be avoided due to off-flavor formation, a mild oxidation reaction is sometimes desirable for the development of characteristic nut volatile odors. Frequently, equilibrium among them is important to define the characteristic volatile profile of a nut [8]. Additionally, it is important to know if there are differences in key odorants among different types of nuts, but also how the processing methods affect the volatile profiles. Finally, a discussion on the most extended analytical methods employed for volatile determination and a brief recompilation of sample preparation and quantification variants will be conducted.

## 2. Main Volatile Organic Compounds (VOCs) Present in Nuts

**Figure 3:**
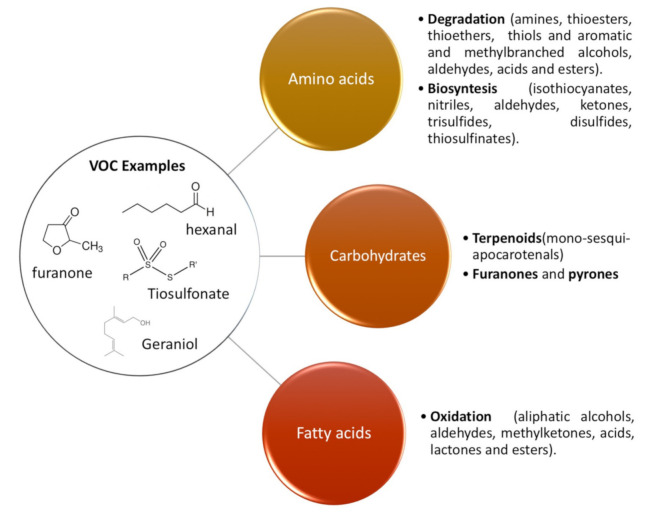
Scheme showing different pathways from the main precursors of nut volatiles [3,10].

The final flavor of nuts is conditioned by the volatile compounds generated during fruit growth and maturation. Further changes in the volatile profile can occur during the storage after the harvesting of fruits and in the processing and cooking of nuts, thus affecting their sensorial quality [9]. The precursors of the VOCs are mainly fatty acids, carbohydrates and amino acids present in the plants and fruits (Figure 3). Among the VOCs in nuts, it is possible to find saturated and unsaturated molecules with straight, branched or cyclic structures including different functional groups such as alcohols, aldehydes, ketones, esters and ethers and also nitrogen and sulfur [10].

The most representative volatile compounds derived from fatty acid oxidation are aldehydes and alcohols [11]. The concentration level of some of these compounds can be employed to indicate the degree of oxidative deterioration of a food sample. In this sense, in walnut oils, the main ones associated with rancid flavor were 2-octenal, hexanal, 2-heptenal, 1-octen-3-ol, hexanoic acid and nonanal, whereas in almond oils, the total oxidation of lipids was mainly related to 1-pentanol, hexanal, and hexanoic acid [12]. In relation to peanuts, it was found that the compounds that were directly linked to an oxidized flavor were octanal, nonanal, hexanal and 2-pentylpyridine [13]. Hexanal is the most characteristic volatile product formed as a consequence of nut oxidation due to the high concentration of linoleic acid in the fat of the nuts. Another important aldehyde is nonanal, which appears as a by-product of oleic acid degradation [14]. Moreover, the most relevant volatile compounds present in the aroma notes of hazelnuts are in the group of aldehydes such as octanal (soapy), hexanal (grassy or green) and 2-methybutanal (malty). As alcohols, linalool is representative of flowery odor and ketones such as 2-hepten-4-one and 5-methyl-(Z)-hepten-4-one are responsible for nutty and fruity odors and 2,3- butanedione of buttery. Other important volatiles in hazelnuts are pyrazines such as 3,6-dimethyl-2-ethyl pyrazine (roasted and earthy) among other compounds [8].

Some odor notes in nuts are associated with unripe fruits, which is mainly related to hexane derived molecules such as hexanal, cis-3-hexanal, and hexanol, among others, their precursors being linolenic and linoleic fatty acids [15]. The same fatty acids are also precursors of other volatile compounds formed after decarboxylation of the alfa-keto acids to generate aldehydes such as 3-methylbutanal and 2-methylbutanal and other respective alcohols. Additionally, free aromatic amino acids such as phenylalanine can suffer decarboxylation processes and some precursors of floral aromas are formed such as 2-phenylethanol and phenylacetaldehyde [16].

Meanwhile, terpenoids are also present in the volatile profile of nuts, providing the characteristic aroma of fresh nuts. These compounds are molecules with an isopentane skeleton such as valencene, limonene, and linalool. In this sense, linalool is also responsible for the flavor of a lot of fruits such as tomatoes and oranges and some herbs such as lavender and basil [3]. Finally, other components such as carotenoids are also precursors of some fruity and floral smells formed during the maturity stage. Some examples of these compounds are ketones such as 6-methyl-5-hepten-2-one and β-ionone.

## 3. Analysis of VOCs Present in Nuts

The large number of volatile compounds, usually up to 200, that can be identified in nuts, make their identification and quantification difficult [12]. In this case, a separation technique such chromatography is usually employed. The volatile nature of the compounds determined makes gas chromatography (CG) coupled to mass spectrometry (MS) the preferred chromatographic type utilized for this analysis [17]. By using this technique, volatile compounds are commonly introduced in the chromatograph by using the split or splitless mode but if a higher sample capacity is needed due to a low volatile concentration in the sample, a programmed temperature vaporizer (PTV) is employed as an injection port [18]. In order to identify the main VOCs, the most extended stationary phases are nonpolar phases such as polydimethylsiloxane, columns with 5% phenyl groups or moderately polar phases such as polyethylene glycols. The length of the employed columns can vary between 25 and 30 m, but if complex mixtures of volatile compounds need to be separated, the length of the column must be increased up to 200 m [18]. Finally, the detector selected is usually a mass spectrometer that allows spectral information of the peaks to be obtained for identification of the target analytes. In routine analysis, the flame ionization detector (FID) can also be employed [18].

Prior to the identification and quantification of volatiles in nuts, the extraction of the target compounds must be optimized. A reference method for the extraction of VOCs in food matrices is liquid extraction (LE) with organic solvents. This methodology implies the employment of great amounts of organic solvents and, in many cases, low recoveries are obtained [19]. To solve this problem, liquid–liquid microextraction (LLME), dispersive liquid–liquid extraction (DLLE), stir bar sorptive extraction (SBSE) and solid-phase microextraction (SPME) are also employed. All of these analytical methodologies are commercially available, and extensively used.

SPME is nowadays the most commonly used extraction methodology for the analysis of volatile compounds in food samples, either in direct or in headspace (HS) mode, since it is a solvent-free extraction process that employs lower amounts of sample [17]. The analytical methodology consists of a fiber holder, in which a fiber coated with polymeric material is employed to retain the volatiles. After this, the analytes can be directly desorbed on the injection port of a chromatograph [20]. SPME methodology involves equilibration and extraction steps. Firstly, the sample inside a sealed vial is exposed to a selected temperature for a specific period of time in order to promote analyte volatilization into the HS in the equilibration step. Secondly, the coated fiber is immersed in the HS of the sample, maintaining a constant temperature, for a selected period of time to extract the target compounds. Experimental conditions (time and temperature values) of both steps, equilibration and extraction, commonly employed in VOCs analysis in nut samples are depicted in Table 1.

SPME results depend on the quantity of sample, and time and temperature extraction values. The influence of both factors—time and temperature—should be analyzed simultaneously since a temperature increase induces a drop in process time in many cases. Consequently, it is reported that higher temperatures reduce the needed equilibration time [21]. However, shorter equilibration times produced higher relative standard deviations (RSD) [19]. Pastorelli et al. tested higher temperatures (50, 60, 70 and 80 °C) and short equilibration times (5–60 min) [15]. Optimal values (60 °C, 10 min) proved a remarkable time reduction at higher temperatures. In addition, ultrasonic extraction has been used in place of the extraction by using temperature for analyzing the volatile compounds present in almonds [22].

Meanwhile, the sample is often agitated using a stirring bar to decrease the time necessary for equilibration [23]. However, as is shown in Table 1, a steady extraction process is commonly used as well. In fact, Pillonel et al. demonstrated that stirring has a minimal effect in the extraction yield of highly volatile compounds [24]. In addition, the solid nature of nut samples makes their agitation difficult, and the addition of water is necessary for homogenization purposes [22]. Moreover, some researchers increased the medium ionic strength in order to modify the nature of the matrix and the extraction efficiency, since this addition could affect the partition coefficient of the analytes [25,26]. Nevertheless, the higher the ionic strength, the lower the solubility of neutral molecules in water and the less likely these molecules are to pass from the solid matrix to the water. As a result, the extraction efficiency of these molecules decreases [19]. Consequently, most authors decided to avoid the addition of salt [19,27,28,29]. It is interesting to highlight that no water addition was required when hexanal was the only target compound [15]. Sample amount is also a key factor in the extraction process of volatile compounds in nuts [15]. As can be seen in Table 1, different sample amounts have been tested, most of them being higher than 0.1 g.

Finally, analyte extraction depends on fiber type. The commercially available fiber materials are carboxen (CAR), polydimethylsiloxane (PDMS) and divinylbenzene (DVB) [21]. In order to choose the most suitable extraction phase composition, the highest affinity of the target analytes is the key factor in the extraction efficiency. The vast majority of authors opt to employ three-phase CAR/PDMS/DVB fiber because of its high extraction capacity, which allows the removal of a high number of odorants [19]. Nevertheless, depending on the target compound, other coatings could also be better for the extraction process. As an example, Pastorelli et al. showed that CAR/PDMS fiber had higher sensitivity for hexanal extraction than PDMS, PDMS/DVB, CAR/DVB, and DVB/PDMS/CAR fibers [15].

Once the results are obtained, some authors directly employ the area of the compound [19,30,31] (or the percentage of areas [32,33,34]) if the analysis has the aim of comparing different varieties or the processing conditions of nuts. However, in some studies, a complete quantification was performed. Different types of quantification approaches are shown in Table 1. Most researchers employ semi-quantification with internal standard (IS), which is described in Equation (1), Equation (2) being an extension of the previous one. In both cases, the procedure is simple due to the fact that an external standard (ext. std.) curve is not required. Actual quantification is depicted in Equation (3). External standards are commonly prepared in deionizer water [35] or in organic solvents (e.g., n-hexane [25]).

(1) $$\mathrm{Concentration}=\frac{\mathrm{Extraction}\;\mathrm{ion}\;\mathrm{peak}\;\mathrm{area}}{\mathrm{Extracted}\;\mathrm{ion}\;\mathrm{peak}\;\mathrm{area}\;\mathrm{of}\;\mathrm{IS}}\left[\mathrm{IS}\right]$$

(2) $$\mathrm{Concentration}=\frac{\left(\mathrm{Extracted}\;\mathrm{ion}\;\mathrm{peak}\;\mathrm{area}\right)/\left(\mathrm{Extracted}\;\mathrm{ion}\;\mathrm{peak}\;\mathrm{area}\;\mathrm{of}\;\mathrm{IS}\right)}{\mathrm{Sample}\;\mathrm{weight}}\left[\mathrm{IS}\right]$$

(3) $$\mathrm{Concentration}=\frac{\mathrm{Extracted}\;\mathrm{ion}\;\mathrm{peak}\;\mathrm{area}}{\mathrm{Slope}\;\mathrm{of}\;\mathrm{Ext}.\mathrm{Std}.\mathrm{curve}}$$

Franklin et al. prepared external standards in a devolatized nut matrix, adding a mix solution of deuterated analytes [12,21]. Deuterated compounds were employed as internal standards. Octanal-d16, 2-methylpyrazine-d6, and n-hexyl-d13 alcohol were used as internal standards to determine aldehydes, pyrazines, and alcohols, respectively [36,37,38]. Octanal, 2-methylpyrazine and 1-hexanol were also employed for this purpose [22]. However, other studies used only a compound as the internal standard such as 4-methyl-2-pentanone [2,39] or 2-pentanol in pistachios [28].

Additionally, it is important to keep in mind that there are great differences in the perception threshold concentrations in humans for the different volatile compounds found in nuts. Based on this, to identify the most relevant compounds present in the sensorial profile of nuts, sometimes the odor activity value (OAV) is referenced. This value is calculated as the ratio between the concentration of a volatile compound present in a sample and the perception threshold concentration of the same compound. Volatiles with OAV values higher than 1 are detectable by consumers and, for this reason, are considered key odorants. Chetschik et al. investigated the volatile profile of peanuts and 26 compounds were quantified, but only 11 of them presented OAVs higher than 1 [40]. The compounds with a higher impact on the overall flavor of raw peanuts were 3-isopropyl-2-methoxypyrazine, acetic acid, 3-methylthiopropanal, 2,3-pentanedione and hexanal [40]. Meanwhile, 5-methyl-4-heptanone and 2-methoxy-3,5-dimethylpyrazine were the volatiles with higher OAVs present in raw hazelnuts [41]. On the other hand, 2,3-pentanedione, methional, 2-acetyl-1-pyrroline and 2-Acetyl-3,4,5,6(or 1,4,5,6)-tetrahydropyridine were detected in raw almonds. However, OAVs for raw almonds were significantly lower than the ones obtained for the major representative volatiles in peanuts and hazelnuts [42].

Nevertheless, when thermal treatment is applied to nuts, the OAV profile changes, and a higher number of compounds with values greater than 1 appears. The peanut’s key odorants, responsible for the typical roasted notes, are methanethiol followed by 2,3-pentanedione. Furthermore, some products of the initial steps of the Maillard reaction appeared with high OAV values such as 3-methylthiopropanal, 2-acetyl-1-pyrroline, 3-methylbutanal, and 2-methylbutanal [40]. In the case of pan-roasted hazelnuts, the volatile compounds which presented the highest OAV values resulted to be 3-methylbutanal, followed by distance by 4-hydroxy-2-5-dimethyl-3(2H)-furanone, (E,E)-2,4-decadienal, nonenal, hexenal and octanal [41]. Additionally, in the case of dry- or oil-roasted almonds, the principal biomarkers were 2,3-pentanedione, methional, followed by 2-acetyl-1-pyrroline and 4-hydroxy-2,5-dimethyl-3(2H)-furanone [42], even though other compounds in lower quantities could also be used as biomarkers.

## 4. Effect of Harvesting Conditions and Healthy State of Nuts on Volatile Profile

The main volatile compounds reported in raw nuts are reviewed in Table 2. As expected, several differences are detected that could be related to nut composition. Additionally, different factors could change the characteristic volatile profile of raw nuts. In this section, the effect of harvesting conditions is reviewed as well as the healthy state of nuts.

The current limitation of water resources is threatening nut productivity, so new sustainable agronomic practices, such as regulated deficit irrigation (RDI), have been implemented. These irrigation strategies do not affect fruit quality [46,47]; however, water stress during the growing of nuts can modify its volatile profile [48]. For example, almonds produced under an RDI strategy presented higher total volatile content. In a study with pistachios, it was found lower water stress conditions produced nuts with higher terpene content such as α-pinene [49,50]. This compound is the most important volatile compound in pistachio samples, and it was observed that weather can influence the biosynthesis of VOCs [51] and also the harvesting time [23,25]. In another study, it was also evidenced that the flavor of pistachios was minimized by irrigation [49].

Moreover, the volatile organic profile of nuts could be modified by the state of health of the nut. The navel orangeworm (NOW) is among the major concerns in the almond industry, due to it being able to cause fungal infection. VOCs emission of almonds and their relationship with NOW have been investigated [52]. Although differences in the volatile profile have been found, the identification of particular compounds and their relationship to NOW have not been addressed. Mature almonds from the Monterey variety were evaluated for their volatile composition after mechanical damage and compared with the volatile composition of undamaged almonds. 3-pentanol and two isomers of a spiroketal chalcogran were found in the damaged almonds. Moreover, the concentration of some compounds such as a spiroketal conophthorin, numerous four-carbon esters and ketones as well as alcohol derivatives, in addition to two eight-carbon chain compounds, increased in the damaged almonds [52,53].

Changes in volatile organic compounds have been evaluated as an indicator of aflatoxin contamination [31,54,55]. Beck et al. studied the volatile emissions of whole and blanched almonds naturally contaminated with aflatoxins. Volatiles indicative of fatty acid decomposition were predominant in the samples that underwent some form of blanching. Moreover, they found an increase in the concentration of some aldehydes (e.g., hexanal, heptanal, octanal) and hexanoic acid [56]. A similar study was carried out with pistachios. A comparison of volatile compounds in healthy and naturally or artificially aflatoxin-contaminated pistachios was carried out by Georgiadou et al. They found some differences in specific compounds such as C-8 alcohols and aldehydes, sesquiterpenes, and monoterpenes, among others. These compounds allowed differentiation among contaminated and healthy pistachios by applying principal component analysis [28].

## 5. Volatile Profile of Nuts after Thermal Treatments

Processed nuts are consumed as a snack or added to confectionary and bakery products. For this purpose, heat treatments such as roasting and frying are necessary during nut processing to improve their sensory quality, digestibility, and microbiological safety. These heat treatments may significantly affect their properties and quality attributes, obtaining appreciable and desired changes in their texture, color, flavor and taste [58]. Interestingly, when nuts are thermally treated, new VOCs are formed from different reactions that are produced, others disappear, and others increase. Knowledge of the key odorants in thermal treatments of nuts can help to select the best preservation and thermal processing conditions. In this section, the most popular thermal processing methods of nuts and their influence on volatile profile are reviewed (Table 3).

### 5.1. Roasting Thermal Processing

The most popular thermal processing method of nuts is hot air roasting. During this process, samples are heated to temperatures of 130–200 °C from 5 to 60 min [69]. As a result, different reactions develop, which affects the nut’s volatile profile. These main reactions are the Maillard reaction, caramelization, fatty acid autoxidation, lipid degradation and the degradation of sulfur-containing amino acids.

The roasting process is associated with non-enzymatic darkening reactions such as, for example, the Maillard and caramelization reactions. The Maillard reaction initiates when amino acids and certain reducing sugars react and produce diverse kinds of compounds depending on the reactants, but also on the pH and the temperature reached during the roasting process. In the Maillard reaction, some heterocyclic volatile compounds such as furans, ketones, pyrazines, pyrroles, aldehydes and pyridines are formed and some of them show an increase compared to raw homologue nuts [62]. As shown in Table 3, some specific compounds related to this reaction in nuts are benzyl alcohol, 2,3-pentanedione, furfural, phenylacetaldehyde, toluene, 2,5-dimethyl-4-hydroxy-3(2H)-furanone, 2-furfurylthiol and some nitrogen-containing compounds including pyrazines such as 2,5-methylpyrazine, 2-methylpyrazine, dimethylpyrazine and trimethylpyrazine [36,59,61]. Some reported compounds are formed by the Strecker degradation reaction of amino acids in the Maillard reaction such as 2-methylbutanal, 3-methylbutanal and 2-acetyl-1-pyrroline [42]. Benzaldehyde is produced from phenylalanine under heat [61]. Benzeneacetaldehyde is a compound formed from phenylalanine by the action of polyphenol oxidase, and has been found in freshly roasted almonds, with honey-like scent, harsh, and hawthorn odor descriptors [22].

One important reaction is the degradation of sugars in the caramelization process [42]. In this process, heterocyclic oxygen furans are formed such as furfural. Another important reaction is fatty acid autoxidation. Hexanal and 2-nonenal, among others, are products of the degradation of linoleic acid; meanwhile, for example, nonanal and 2-decenal come from oleic acid [61]. Moreover, lipid degradation is a very important process for the volatile profiles of nuts, in which some aldehydes, alcohols and ketones are formed, being responsible for the desire toasted flavor [42]. Alcohols were also generated through lipid degradation such as 1-octen-3-ol, which comes from the thermal decomposition of methyl linoleate hydroperoxide, and contributes to a herbaceous aroma, whereas 1-butanol is formed from the decomposition of linolenic acid and is responsible for an unripe apple aroma [61]. Regarding ethanol, it was at high concentration among the volatile compounds in roasted cashew nut, which contributes to the formation of esters that usually have fruity odors [65]. Acetone found in roasted cashew is a result of the reaction of D-xylose with valine, while heptane does not contribute to aroma [65].

Sulfur-containing compounds also increase during the roasting process because of the degradation of sulfur-containing amino acids, such as cysteine and methionine. Sulfur compounds such as dimethyl sulfide give a taste of fresh onions to the oils. In relation with terpene compounds, in a study with pistachios, it was found that VOCs analysis shows that Argentinean pistachio nuts are rich in monoterpenes, mainly limonene (citrus, mint), α-pinene (pine, turpine), and 3-carene (lemon, resin) [64]. According to the mentioned work, it is possible to define the limonene as a marker for unroasted pistachio, while α-pinene and 3-carene seem to be associated with roasted pistachio with and without salt. In this sense, the perception of roasting is associated with increased amounts of α-pinene and 3-carene in roasted pistachio, while the raw sample is associated with higher amounts of limonene. Finally, it seems that any difference was reported due to the addition of salt.

To sum up, changes in the volatile profile due to the roasting treatment significantly affect the quality attributes of nuts, obtaining appreciable and desired changes in flavor. For example, the presence of 4-hydroxy-2,5-dimethyl-3(2H)-furanone in roasted hazelnuts was detected at high concentrations. This compound is formed after the dehydration of reducing monosaccharides. The changes in concentration of this volatile compound explain the differences in sensorial perception (popcorn-like, coffee-like and caramel) after eating roasted hazelnuts; meanwhile, in raw hazelnuts, the perception is associated with a fruity and nutty aroma [59].

### 5.2. Novel Roasting Treatments

Although hot air roasting is the most popular heat treatment of nuts, this operation is time and energetically expensive. As a consequence, new processing methods have been applied to reduce these drawbacks.

In this context, microwave energy offers the advantages of speed of operation, energy savings, precise process control, and faster start up and shut-down times, compared with conventional heat processes. Microwaves have been used in the roasting of almonds [61], and pistachios [66]. Regarding almonds, microwave heating enhanced the production of volatiles compared to frying and air-hot heating, and the flavor was the most preferred by panelists. Although the main VOCs were similar to those obtained by hot air roasting, two additional compounds were reported. On the one hand, the Strecker aldehyde methional contributes vegetable and creamy aromas. On the other hand, the undecane and 1,4-butyrolactone were obtained from the lipid oxidation of the nut—the lactone contributes to milky and creamy aromas. With respect to pistachios, Hojjati et al. [66] found that limonene, nonanal, and α-pinene were the main components in microwave-roasted pistachios. In this sense, the treatment variables, i.e., time and microwave power, affected the volatile concentrations of the nut. In particular, the total concentration of volatiles increased with the time and power of microwave roasting.

Radio frequency is a novel thermal processing technology with a frequency range of 3 kHz–300 MHz. Heat is generated inside the food products by molecular friction, owing to ionic conduction and dipole rotation during heating. Compared with microwave heating, radio frequency has a deeper penetration depth and a relatively uniform electromagnetic field distribution, which allows the bulk and thick materials to be heated more efficiently [67]. Recently, a roasting processing method of almonds has been reported by using the radio frequency technique for 15 min at 120–130 °C. As a result, similar major volatile compounds were obtained. Moreover, radio frequency roasting produced almonds with desired roasted flavor compounds at a lower roasting temperature.

### 5.3. Deep-Frying Processing

During the deep-frying process, nuts are immersed in different frying oils at 160–200 °C in the presence of air [69]. Frying involves the immersion of food in hot oil and during this process, some of the moisture is replaced by the frying oil, leading to the formation of pores that allow oil penetration into the created voids [70]. As a consequence, it is expected that differences in the volatile profile and sensory characteristics of nuts could appear. Aldehydes, pyrazines and alcohols were the predominant VOCs present in fried almonds [61,62]. Concerning Maillard products, benzyl alcohol, methional, 2-methylbutanal, 3-methylbutanal and 2,5-dimethylpyrazine were reported, whereas 1-butanol, 1-octen-3-ol, hexanal and 1-pentanol were produced due to lipid oxidation. In this sense, the concentration of 1-pentanol increased as a linoleic acid oxidation product [62]. When the results obtained by the frying process were compared with those obtained by hot air roasting, common obtained VOCs were observed, but fried almonds showed 18 additional volatile compounds (2-ethyl-5-methylpyrazine, 3-ethyl-2,5-dimethylpyrazine, diacetyl, (Z)-1,5-octadien-3-one, acetic acid, (E,E,Z)-2,4,6-nonatrienal, cyclotene and 11 unknown compounds). Meanwhile, nine other compounds were identified in the hot air-roasted almonds and not in the fried ones ((E,Z)-2,6-nonadienal, (E)-2-decenal, (E)-4,5-epoxy-(E)-2-decenal, methyl propyl disulfide and five unknown compounds). In general, fried almonds presented a higher number and a higher concentration of furanones and nitrogen-containing compounds. On the other hand, the hot air-roasted almonds were richer in aldehydes and sulfur compounds. The authors justified this behavior as the differences in energy transference and time and temperature. In this sense, in the fried almonds, the energy transfer is mainly produced by conduction, while in hot air-roasted samples, air convection predominates. As oil heat transfer is more efficient, more Maillard reaction and sugar degradation products are formed [42].

Regarding fried chestnuts, hexanal, octanal, nonanal, furfural, 3-heptanone, and 4-hydroxy-2-butanone were identified as the main VOCs after 15 min of processing at 240 °C [68]. In particular, 3-heptanone and 4-hydroxy-2-butanone were underlined as characteristics of the frying thermal processing of chestnuts.

## 6. Conclusions

Although the number of compounds present in the volatile profile of nuts is quite high, some conclusions can be drawn from the review of the published bibliography. The volatile profile of nuts depends both on the chemical composition of the nut in question and on the state of maturity of the product. In this respect, besides terpenoids and substances derived from certain amino acids, volatiles originating as a result of the evolution (i.e., oxidation) of the fatty acids present in the fat fraction of the nut are compounds usually present in the volatile profile of nuts. It is important to mention that the volatile profile may be modified depending on the agronomic conditions of nut production and the presence of microorganisms.

For the determination of volatiles, SPME combined with GC–MS is the most frequently used analytical technique. Optimization of the extraction conditions is one of the most critical aspects of the analysis, since the diversity of compounds present hampers simultaneous optimal conditions for all compounds being found. For the same reason, quantification of the analytes is complicated by using external standards and internal standardization.

It is important to mention than some nuts are consumed after having undergone heat treatment. This treatment can have a significant influence on the volatile profile of the product as a result of the reactions that occur during processing. These reactions are the Maillard reaction, caramelization, oxidation of fatty acids and the degradation of sulfur-containing amino acids. The time and duration of the treatment, as well as the use of a lipid medium for heat transmission, cause the appearance of new compounds and the disappearance of compounds initially present in the nut.

Finally, it should be noted that the determination of the volatile profile of nuts is a complex task but offers many possibilities to control different aspects of food production and evolution.

## Figures and Tables

**Table 1:** Experimental SPME conditions (time and temperature values) of both steps, equilibration and extraction, commonly employed in VOCs analysis in nut samples.

				Equilibration Conditions		Extraction Conditions				
Type of Nut	Sample Amount (g)	Fiber	Agitation	Time (min)	Temperature (°C)	Time (min)	Temperature (°C)	Quantification	Column	Ref.
Almond	5.0 *	1-cm 50/30 µm DVB/CAR/PDMS	No	40	24	30	24	(1)	DB-Wax (30 m × 0.25 mm × 0.25 μm)	[38]
Almond	5.0 *	1-cm 50/30 µm DVB/CAR/PDMS	Yes	45	40	45	40	(3)	DB-Wax (30 m × 0.25 mm × 0.25 μm)	[21]
Almond	0.250 *	1-cm 50/30 µm DVB/CAR/PDMS	No	15	25	30	25	(2)	DB-Wax (30 m × 0.25 mm × 0.25 μm)	[37]
Almond	5.0 *	1-cm 50/30 µm DVB/CAR/PDMS	Yes	45	40	45	40	(3)	DB-Wax (30 m × 0.25 mm × 0.25 μm)	[12]
Almond	3.0 *	1-cm 50/30 µm DVB/CAR/PDMS	No	10	40	30	40	(1)	TRB-5MS (30 m × 0.25 mm × 0.25 μm)	[22]
Beechnut, hazelnut, pistachio and walnut	10.0 **	1-cm 50/30 µm DVB/CAR/PDMS	Yes	60	25	60	25	No	RTx-5 (60 m × 0.25 mm × 0.25 μm)	[30]
Hazelnut	0.1 *	1-cm 75 µm CAR/PDMS	No	10	60	10	60	(3)	DB-Wax (30 m × 0.25 mm × 0.5 μm)	[15]
Hazelnut	1.5 *	2-cm 50/30 µm DVB/CAR/PDMS	No	20	50	20	50	(3) º	MEGA-WAX™ (30 m × 0.20 mm × 0.20 μm)	[43]
Peanut	5.0 *	PDMS/DVB	No	30	60	15	60	(3)	DB-5	[35]
Peanut	5.0 *	50/30 µm DVB/CAR/PDMS	Yes	1440	25	20	21	(1)	SUPELCOWAX™ 10 (30 m, 0.25 mm, 0.25 mm)	[25]
Peanut	3.0 **	1-cm 65 µm PDMS/DVB	Yes	10	50	40	50	(1)	DB-Wax (30 m × 0.25 mm × 0.25 μm)	[44]
Peanut	5.0 **	2-cm 50/30 µm DVB/CAR/PDMS	No	30	80	10	80	(1)	RTX-5MS (30 m × 0.25 mm × 0.25 μm)	[39]
Peanut	5.0 **	1-cm 50/30 µm DVB/CAR/PDMS	Yes	30	50	30	50	No	DB-17MS (60 m × 0.25 mm × 0.25 μm)	[32]
Peanut	0.2 *	2-cm DVB/CAR/PDMS	Yes	8 h	20	50	60	(1)	dB5-MS semi-polar (60 m × 0.32 mm × 1 μm)	[26]
Peanut	5.0 **	1-cm 65 µm DVB/CAR/PDMS	No	20	80	60	80	No	HP-5 (30 m × 0.25 mm × 0.25 μm)	[33]
Pistachio	15.0 *	50/30 µm DVB/CAR/PDMS	Yes	15	50	120	50	No	HP-5 (30 m × 0.32 mm × 0.25 μm)	[19]
Pistachio	8.0 *	50/30 µm DVB/CAR/PDMS	No	-	-	60	83	No	HP-5MS (30 m × 0.25 mm × 0.25 μm)	[34]
Pistachio	10.0 *	50/30 µm DVB/CAR/PDMS		15	50	120	50	(1)	Equity-5 (30 m × 0.25 mm × 0.25 μm)	[28]
Pistachio	24.5 *	PDMS-DVB	No	30	30	20	30	No	Agilent DB-1 (60 m × 0.320 mm × 0.25 μm)	[31]
Pistachio	1.5 **	2-cm 50/30 µm DVB/CAR/PDMS	Yes	-	-	30	40	(1)	DB-Wax (30 m × 0.25 mm × 0.25 μm)	[2]
Walnut	0.5 *	50/30 µm DVB/CAR/PDMS	Yes	15	50	30	60	(1)	RTX-5MS (30 m × 0.25 mm × 0.25 μm)	[27]
Walnut	3.0 ** (mL)	1-cm 50/30 µm DVB/CAR/PDMS	No	10	50	30	50	No	HP-INNOWAX (30 m × 0.25 mm × 0.25 μm)	[45]
Walnut	1.0 **	65 µm PDMS/DVB	No	-	-	30	50	No	CP-Wax52CB (30 m × 0.25 mm × 0.25 μm)	[20]
Pecan	2.0 *	50/30 µm DVB/CAR/PDMS	Yes	30	25	30	65	(1)	HP-5 (30 m × 0.25 mm × 0.25 μm)	[36]

Sample pre-treatment: * Only grinding ** Grinding and oil extraction. Quantification: 1: Semi-quantification with IS, 2: (1) corrected by sample amount, 3: quantification. º: IS was employed. DVB: Divinylbenzene, CAR: Carboxen, PDMS: Polydimethylsiloxane.

**Table 2:** Main VOCs of raw nuts.

Nut	Main VOCs	Ref.
Almond	1-Hexanol, 3-methyl-1-butanol, nonanal, 2-methyl-1-propanol, 1-propanol	[37]
	Benzaldehyde, hexanal, 1,2-propanediol, 1-chloro-2-propanol, 3-methyl-1-butanol, pentanal, 2-heptanone, 1-hexanol.	[38]
	Hexanal, 3-methyl-1-butanol, benzaldehyde, heptanal, nonanal, 1-octanol, 2-octanone	[22]
Chestnut	ϒ-terpinene, phenylaldehyde, hexanal, furfural, α-terpinene	[57]
Hazelnut	α-tujone, β-tujone, 2-pentanone, acetic acid, 3-methyl-2- butanol, n-decane	[43]
Peanut	Hexanoic acid, 2-ethyl-1-hexanol, 1-hexanol, pentanal, hexanal, palmitic acid, 2-ethyl-5-methylpyrazine, heptanal	[44]
	2-Propanone, α-pinene, benzene, α-terpinolene, hexanal, d-limonene	[29]
	Toluene, α-limonene, γ-terpinene, p-cymene, nonanal, β, pinene, hexanoic acid	[39]
	2,5-Dimethylpyrazine, nonanal, hexanal, 2-ethyl-5-methylpyrazine, octanal, 2,5-dimethyl-3-ethylpyrazine	[33]
	Hexanal, benzaldehyde, benzenacetaldehyde, 2,5-dimethylpyrazine, 2-heptenal, 2-ethyl-5-methylpyrazine, trimethylpyrazine, 3-ethyl-2,5-dimethylpyrazine	[26]
Pistachio	9-Octadecenoic acid, α-pinene, 1-methyl-1H-pyrrole, α-terpinolene, limonene, dimethyl-2H-pyran-2-one, 2-octenal, 2-hexenal	[34]
	α-Pinene, α-terpinolene, 1H-pyrrole, ethyl-alcohol, limonene, hexane	[28]
	α-Pinene, β-pinene, 2-ethyl-1-hexanol, α-terpineol, camphene, hexanoic acid	[2]
Walnut	Hexanal, hexanoic acid, 1-pentanol, 2-octenal, pentanal, 2-pentylfuran, propanoic acid	[45]
	2-Octenal, hexanoic acid, hexanal, 2-decenal, 1-octen-3-ol, nonanal	[20]

**Table 3:** Most popular thermal processing methods of nuts and main VOCs reported in the literature.

Thermal Processing	Nut	Processing Conditions	Main VOCs	Ref.
Hot-air roasting	Hazelnut	34, 18, 13 min at 130, 140 and 150 °C	2,3-pentanedione, 2-acetyl-1-pyrroline, dimethyl sulphide, 2-furfurylthiol, 3-methylbutanal, 2-nonenal, 2-decenal, hydroxy-2,5-dimethyl-3(2H)-furanone	[59]
		20, 25 and 30 min at 160 °C	2-methylpropanal, 2-methylbutanal, 3-methylbutanal, 2,5-methylpyrazine	[60]
		40 min at 140 °C	2-methylbutanal, 3-methylbutanal, 2,5-methylpyrazine, furfuryl alcohol, 2-methylpropanal, ehtyl acetate, 2,3-pentanedione, 2-methylpyrazine, 2,5-methylpyrazine, furfural, 1-hydroxy-2-propanone	[43]
	Almond	5 min at 177 °C	Benzyl alcohol, benzaldehyde, 1-octen-3-ol, toluene, dimethylpyrazine, 1-butanol, hexanal	[61]
		5–10 min at 170–190 °C	Hexanal, 2-methyl-butanal, 2-methyl-pyrazine, 2,5-dimethyl-pyrazine, furfural	[62]
		10 min at 190 °C	2,5-dimethyl-pyrazine, trimethylpyrazine	[48]
		33 min at 138 °C	Hexanal, benzeneacetaldehyde, 2,5-dimethyl-pyrazine, nonanal	[38]
		28 and 38 min at 138 °C	2-methylbutanal, 3-methylbutanal, hexanal, benzaldehyde, furfural, 2-phenyl acetaldehyde	[38]
		28, 33 and 38 min at 138 °C	2-methylbutanal, 3-methylbutanal, hexanal, benzaldehyde, furfural, 2-phenyl acetaldehyde	[63]
	Chestnut	25 min at 200 °C	Hexanal, butylacetate, ethylbenzene, 2-hydroxy-2-cyclopenten-1-one	[63]
	Pistachio with and without salt	90 min at 120 °C	α-pinene, limonene, 3-carene	[64]
	Cashew	3 and 9 min at 143 °C	Methylbutanal, hexanal, acetaldehyde, heptane, ethanol, pentane, acetone	[65]
Microwave roasting	Almond	120 V for 2 min	Benzyl alcohol, methional, benzaldehyde, dimethylpyrazine, nonanal, undecane, 1-octen-3-ol, 1,4-butyrolactone	[61]
	Pistachio	480 or 640 W for 2, 3 and 4 min	α-pinene, limonene, nonanal	[66]
Hot air-assisted radio frequency	Almond	15 min at 120–130 °C	2,5-dimethyl-pyrazine, toluene, hexanal and heptane	[67]
Deep-frying	Almond	5 min at 135 °C	Benzyl alcohol, methional, benzaldehyde, 1-butanol, 1-octen-3-ol	[61]
		10–15 min at 160–200 °C	Hexanal, 2-methyl-butanal, 3-methyl-butanal, 2,5-dimethyl-pyrazine, 1-pentanol	[62]
	Chestnut	15 min at 240 °C	Hexanal, octanal, nonanal, furfural, 3-heptanone, 4-hydroxy-2-butanone	[68]

## Data Availability

Not applicable.
